# 4,4′-(Cyclo­hexane-1,1-di­yl)diphenol methanol solvate

**DOI:** 10.1107/S1600536809000427

**Published:** 2009-01-10

**Authors:** Jun Shuai, Ying Liu, Mo Liu, Dengke Liu

**Affiliations:** a School of Chemical Engineering and Technology, Tianjin University, Tianjin 300071, People’s Republic of China; b Tianjin Institute of Pharmaceutical Research, Tianjin 300193, People’s Republic of China

## Abstract

The title compound, crystallized as a methanol solvate, C_18_H_20_O_2_·CH_3_OH, is an inter­mediate in the synthesis of the anti­lipidemic agent clinofibrate. Mol­ecules are packed together with the methanol solvent molecule *via* two O—H⋯O hydrogen bonds. The third O—H⋯O hydrogen bond is between neighboring 4,4′-(cyclo­hexane-1,1-di­yl)diphenol mol­ecules. The dihedral angle between two benzene rings planes is 81.69 (6).

## Related literature

For details of the anti-lipidemic agent clinofibrate, see: Nishizawa *et al*. (1993 ▶). For the synthesis of clinofibrate, see: Zimmerman *et al.* (1974 ▶). For a similar structure, see: Nassimbeni *et al.* (2007 ▶).
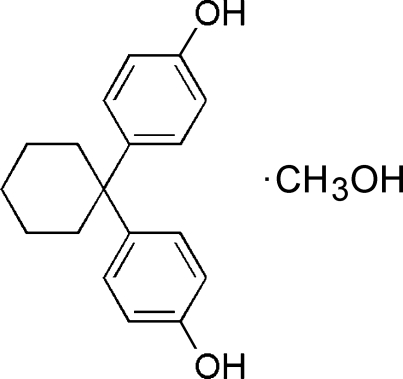


## Experimental

### 

#### Crystal data

C_18_H_20_O_2_·CH_4_O
*M* *_r_* = 300.38
Triclinic, 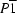

*a* = 6.2245 (12) Å
*b* = 10.889 (2) Å
*c* = 12.712 (3) Å
α = 90.02 (3)°
β = 100.82 (3)°
γ = 90.03 (3)°
*V* = 846.3 (3) Å^3^
*Z* = 2
Mo *K*α radiation
μ = 0.08 mm^−1^
*T* = 113 (2) K
0.16 × 0.12 × 0.08 mm

#### Data collection

Rigaku Saturn diffractometer
Absorption correction: multi-scan (*CrystalClear*; Rigaku/MSC, 2005 ▶) *T*_min_ = 0.988, *T*_max_ = 0.994
8706 measured reflections
2946 independent reflections
2042 reflections with *I* > 2σ(*I*)
*R*_int_ = 0.042

#### Refinement

*R*[*F* ^2^ > 2σ(*F* ^2^)] = 0.058
*wR*(*F* ^2^) = 0.182
*S* = 1.08
2946 reflections
203 parameters
H-atom parameters constrained
Δρ_max_ = 0.33 e Å^−3^
Δρ_min_ = −0.47 e Å^−3^

### 

Data collection: *CrystalClear* (Rigaku/MSC, 2005 ▶); cell refinement: *CrystalClear*; data reduction: *CrystalClear*; program(s) used to solve structure: *SHELXS97* (Sheldrick, 2008 ▶); program(s) used to refine structure: *SHELXL97* (Sheldrick, 2008 ▶); molecular graphics: *SHELXTL* (Sheldrick, 2008 ▶); software used to prepare material for publication: *SHELXTL*.

## Supplementary Material

Crystal structure: contains datablocks I, global. DOI: 10.1107/S1600536809000427/bq2114sup1.cif

Structure factors: contains datablocks I. DOI: 10.1107/S1600536809000427/bq2114Isup2.hkl

Additional supplementary materials:  crystallographic information; 3D view; checkCIF report

## Figures and Tables

**Table 1:** Hydrogen-bond geometry (Å, °)

*D*—H⋯*A*	*D*—H	H⋯*A*	*D*⋯*A*	*D*—H⋯*A*
O1—H1⋯O2^i^	0.82	1.60	2.415 (2)	173
O2—H2⋯O3^ii^	0.82	2.08	2.860 (3)	159
O3—H3⋯O1^iii^	0.82	1.96	2.767 (2)	166

Symmetry codes: (i) 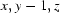; (ii) 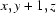; (iii) 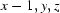.

**Fig. 1.**
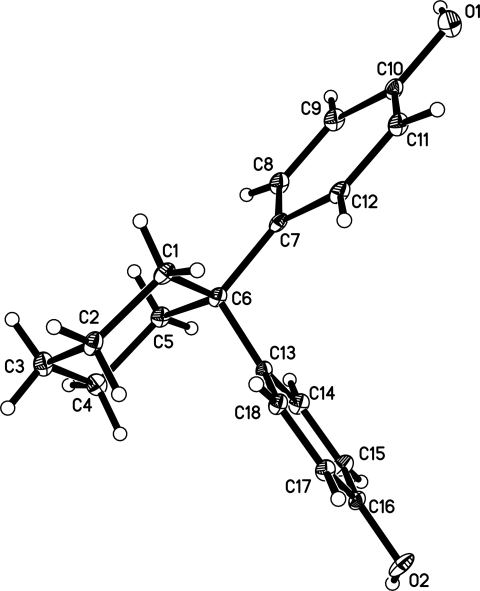
The molecular structure of the title compound with the atom-numbering scheme.

**Fig. 2.**
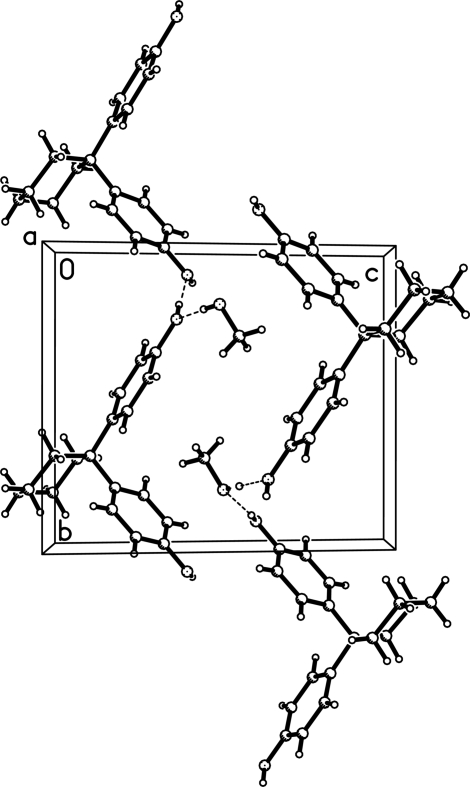
The packing of the title complex. Dashed lines show intermolecular O—H···O hydrogen bonds.

## supplementary crystallographic information

### Comment

Clinofibrate is an anti-lipidemic agent which is effective for the treatment of decreasing Blood lipid (Nishizawa *et al.*, 1993). 4,4'-(cyclohexane-1,1-diyl)diphenol is an important intermediate in the synthesis of clinofibrate (Zimmerman *et al.*, 1974). A similar structure, 4, 4'-(cyclohexane-1,1-diyl)diphenol±3-chlorophenol and 4, 4'-(cyclohexane-1,1-diyl)diphenol±4-chlorophenol (Nassimbeni *et al.*, 2007), has been reported previously. Now we present the crystal structure of the title compound(I). The molecules of (I) are crystallized with the solvent molecule methanol (Fig. 1.). Adjacent molecules of (I) are linked *via* intermolecular O—H···O interactions between the O1—H1 and O2 from a neighboring molecule. The other two H bonds (O2—H2···O3 and O3—H3···O1) are formed between methanol and two neighboring (I) (Table 1.). Planar molecules are usually stabilized by π–π intermolecular interactions. However, (I) is not planar (the dihedral angle between two benzene rings planes is 81.69 (6)°), indicating an absence of π–π coupling.

### Experimental

A mixture of cyclohexanone (196.g, 0.2 mol) and phenol (37,6 g, 0.4 mol) in hydrochloric acid (40 ml) and glacial acetic acid (20 ml) was heated at 328 K for 12 h. The mixture was stirred overnight at room temperature, and the resultant precipitates were collected by filtration. The filtrates were dissolved in acetone (200 ml); the solutions decolorize by active carbon, and concentrated under reduced pressure. The residue was washed by toluene (20 ml) to get white powder. The powder was dissolved in methanol and standing under 277 K, then the white crystals were generated slowly.

### Refinement

All the H atoms was located on their parent atoms with C—H=0.93 (aromatic CH), 0.97 (CH2) and 0.96 (CH3), and O—H=0.82 (*U*_iso_(H)=1.5*U*_eq_(O)), thereafter refined isotropically.

### Crystal data

**Table:**

C_18_H_20_O_2_·CH_4_O	*Z* = 2
*M**_r_* = 300.38	*F*(000) = 324
Triclinic, *P*1	*D*_x_ = 1.179 Mg m^−^^3^
Hall symbol: -P 1	Mo *K*α radiation, λ = 0.71073 Å
*a* = 6.2245 (12) Å	Cell parameters from 2488 reflections
*b* = 10.889 (2) Å	θ = 2.5–27.5°
*c* = 12.712 (3) Å	µ = 0.08 mm^−^^1^
α = 90.02 (3)°	*T* = 113 K
β = 100.82 (3)°	Block, colorless
γ = 90.03 (3)°	0.16 × 0.12 × 0.08 mm
*V* = 846.3 (3) Å^3^	

### Data collection

**Table:**

Rigaku Saturn diffractometer	2946 independent reflections
Radiation source: rotating anode	2042 reflections with *I* > 2σ(*I*)
confocal	*R*_int_ = 0.042
ω scans	θ_max_ = 25.0°, θ_min_ = 2.5°
Absorption correction: multi-scan (*CrystalClear*; Rigaku/MSC, 2005)	*h* = −7→7
*T*_min_ = 0.988, *T*_max_ = 0.994	*k* = −12→12
8706 measured reflections	*l* = −15→14

### Refinement

**Table:**

Refinement on *F*^2^	Primary atom site location: structure-invariant direct methods
Least-squares matrix: full	Secondary atom site location: difference Fourier map
*R*[*F*^2^ > 2σ(*F*^2^)] = 0.058	Hydrogen site location: inferred from neighbouring sites
*wR*(*F*^2^) = 0.182	H-atom parameters constrained
*S* = 1.08	*w* = 1/[σ^2^(*F*_o_^2^) + (0.1196*P*)^2^] where *P* = (*F*_o_^2^ + 2*F*_c_^2^)/3
2946 reflections	(Δ/σ)_max_ = 0.002
203 parameters	Δρ_max_ = 0.33 e Å^−^^3^
0 restraints	Δρ_min_ = −0.47 e Å^−^^3^

### Special details

**Table:**

Geometry. All e.s.d.'s (except the e.s.d. in the dihedral angle between two l.s. planes) are estimated using the full covariance matrix. The cell e.s.d.'s are taken into account individually in the estimation of e.s.d.'s in distances, angles and torsion angles; correlations between e.s.d.'s in cell parameters are only used when they are defined by crystal symmetry. An approximate (isotropic) treatment of cell e.s.d.'s is used for estimating e.s.d.'s involving l.s. planes.
Refinement. Refinement of *F*^2^ against ALL reflections. The weighted *R*-factor *wR* and goodness of fit *S* are based on *F*^2^, conventional *R*-factors *R* are based on *F*, with *F* set to zero for negative *F*^2^. The threshold expression of *F*^2^ > σ(*F*^2^) is used only for calculating *R*-factors(gt) *etc*. and is not relevant to the choice of reflections for refinement. *R*-factors based on *F*^2^ are statistically about twice as large as those based on *F*, and *R*- factors based on ALL data will be even larger.

### Fractional atomic coordinates and isotropic or equivalent isotropic displacement parameters (Å^2^)

**Table:**

	*x*	*y*	*z*	*U*_iso_*/*U*_eq_	
O1	0.6975 (2)	0.24041 (13)	0.37035 (12)	0.0310 (4)	
H1	0.6022	0.1903	0.3764	0.046*	
O2	0.4365 (3)	1.08574 (14)	0.39912 (12)	0.0299 (4)	
H2	0.3199	1.1092	0.4133	0.045*	
O3	0.1059 (3)	0.19930 (17)	0.49467 (14)	0.0468 (6)	
H3	−0.0039	0.2132	0.4495	0.070*	
C1	0.4012 (3)	0.69382 (19)	0.01335 (15)	0.0191 (5)	
H1A	0.3732	0.6162	−0.0242	0.023*	
H1B	0.5580	0.7033	0.0361	0.023*	
C2	0.3017 (3)	0.80790 (19)	−0.06599 (15)	0.0210 (5)	
H2A	0.3550	0.8835	−0.0299	0.025*	
H2B	0.3662	0.8022	−0.1296	0.025*	
C3	0.0496 (3)	0.8225 (2)	−0.10442 (16)	0.0239 (5)	
H3A	0.0202	0.8974	−0.1459	0.029*	
H3B	−0.0058	0.7540	−0.1504	0.029*	
C4	−0.0694 (3)	0.82727 (19)	−0.00891 (16)	0.0219 (5)	
H4A	−0.2261	0.8184	−0.0325	0.026*	
H4B	−0.0401	0.9041	0.0298	0.026*	
C5	0.0328 (3)	0.70978 (18)	0.06654 (15)	0.0199 (5)	
H5A	−0.0402	0.7085	0.1276	0.024*	
H5B	−0.0124	0.6360	0.0255	0.024*	
C6	0.2867 (3)	0.69626 (18)	0.11244 (15)	0.0164 (5)	
C7	0.3890 (3)	0.57198 (18)	0.18020 (15)	0.0168 (5)	
C8	0.2680 (3)	0.48381 (18)	0.19927 (16)	0.0212 (5)	
H8	0.1178	0.4869	0.1743	0.025*	
C9	0.3686 (3)	0.37378 (19)	0.26271 (15)	0.0228 (5)	
H9	0.2704	0.3129	0.2738	0.027*	
C10	0.5952 (3)	0.34952 (19)	0.30856 (15)	0.0205 (5)	
C11	0.7200 (3)	0.43517 (19)	0.29044 (16)	0.0206 (5)	
H11	0.8703	0.4310	0.3149	0.025*	
C12	0.6166 (3)	0.54470 (18)	0.22754 (15)	0.0202 (5)	
H12	0.7154	0.6056	0.2171	0.024*	
C13	0.3242 (3)	0.80415 (17)	0.18613 (15)	0.0161 (5)	
C14	0.2027 (3)	0.83272 (18)	0.26803 (15)	0.0201 (5)	
H14	0.0856	0.7812	0.2726	0.024*	
C15	0.2381 (3)	0.92511 (18)	0.33890 (15)	0.0212 (5)	
H15	0.1514	0.9387	0.3899	0.025*	
C16	0.4000 (3)	0.99216 (18)	0.33016 (15)	0.0186 (5)	
C17	0.5266 (3)	0.96472 (18)	0.25107 (15)	0.0188 (5)	
H17	0.6460	1.0151	0.2481	0.023*	
C18	0.4885 (3)	0.87244 (18)	0.18052 (15)	0.0171 (5)	
H18	0.5767	0.8588	0.1302	0.021*	
C19	0.1690 (4)	0.3024 (2)	0.54959 (18)	0.0327 (6)	
H19A	0.0452	0.3391	0.5725	0.049*	
H19B	0.2282	0.3589	0.5045	0.049*	
H19C	0.2786	0.2830	0.6111	0.049*	

### Atomic displacement parameters (Å^2^)

**Table:**

	*U*^11^	*U*^22^	*U*^33^	*U*^12^	*U*^13^	*U*^23^
O1	0.0309 (9)	0.0222 (9)	0.0363 (9)	−0.0127 (7)	−0.0028 (7)	0.0079 (7)
O2	0.0336 (9)	0.0277 (9)	0.0282 (9)	−0.0155 (7)	0.0057 (7)	−0.0146 (7)
O3	0.0422 (11)	0.0542 (12)	0.0436 (11)	−0.0301 (9)	0.0073 (8)	−0.0248 (9)
C1	0.0167 (10)	0.0204 (11)	0.0205 (11)	−0.0082 (8)	0.0039 (8)	−0.0053 (8)
C2	0.0215 (11)	0.0245 (12)	0.0170 (10)	−0.0090 (9)	0.0039 (9)	−0.0021 (8)
C3	0.0229 (11)	0.0257 (12)	0.0214 (11)	−0.0088 (9)	−0.0004 (9)	0.0027 (9)
C4	0.0142 (10)	0.0233 (12)	0.0266 (12)	−0.0064 (9)	0.0000 (9)	0.0022 (9)
C5	0.0179 (11)	0.0201 (11)	0.0213 (11)	−0.0076 (8)	0.0023 (9)	−0.0008 (8)
C6	0.0139 (10)	0.0164 (11)	0.0185 (11)	−0.0077 (8)	0.0019 (8)	−0.0020 (8)
C7	0.0183 (10)	0.0164 (11)	0.0157 (10)	−0.0083 (8)	0.0035 (8)	−0.0048 (8)
C8	0.0180 (11)	0.0214 (12)	0.0228 (11)	−0.0100 (9)	0.0005 (9)	−0.0016 (9)
C9	0.0196 (11)	0.0212 (12)	0.0263 (12)	−0.0115 (9)	0.0010 (9)	−0.0004 (9)
C10	0.0247 (11)	0.0173 (11)	0.0177 (10)	−0.0077 (9)	−0.0004 (9)	−0.0009 (8)
C11	0.0154 (10)	0.0219 (11)	0.0232 (11)	−0.0081 (9)	0.0005 (9)	−0.0014 (9)
C12	0.0190 (11)	0.0191 (11)	0.0219 (11)	−0.0119 (9)	0.0020 (9)	−0.0042 (8)
C13	0.0159 (10)	0.0165 (11)	0.0149 (10)	−0.0056 (8)	0.0000 (8)	0.0012 (8)
C14	0.0177 (10)	0.0200 (11)	0.0229 (11)	−0.0109 (9)	0.0049 (8)	0.0000 (8)
C15	0.0236 (11)	0.0226 (12)	0.0193 (11)	−0.0103 (9)	0.0091 (9)	−0.0022 (9)
C16	0.0229 (11)	0.0159 (11)	0.0150 (10)	−0.0068 (9)	−0.0011 (8)	−0.0008 (8)
C17	0.0178 (10)	0.0182 (11)	0.0197 (11)	−0.0104 (9)	0.0017 (9)	0.0023 (8)
C18	0.0165 (10)	0.0186 (11)	0.0163 (10)	−0.0069 (8)	0.0033 (8)	−0.0009 (8)
C19	0.0352 (13)	0.0298 (14)	0.0313 (13)	−0.0126 (11)	0.0018 (11)	−0.0088 (10)

### Geometric parameters (Å, °)

**Table:**

O1—C10	1.499 (2)	C7—C8	1.271 (3)
O1—H1	0.8200	C7—C12	1.462 (3)
O2—C16	1.335 (2)	C8—C9	1.513 (3)
O2—H2	0.8200	C8—H8	0.9300
O3—C19	1.341 (3)	C9—C10	1.446 (3)
O3—H3	0.8200	C9—H9	0.9300
C1—C6	1.560 (3)	C10—C11	1.262 (3)
C1—C2	1.647 (3)	C11—C12	1.511 (3)
C1—H1A	0.9700	C11—H11	0.9300
C1—H1B	0.9700	C12—H12	0.9300
C2—C3	1.562 (3)	C13—C18	1.277 (3)
C2—H2A	0.9700	C13—C14	1.431 (3)
C2—H2B	0.9700	C14—C15	1.341 (3)
C3—C4	1.537 (3)	C14—H14	0.9300
C3—H3A	0.9700	C15—C16	1.266 (3)
C3—H3B	0.9700	C15—H15	0.9300
C4—C5	1.653 (3)	C16—C17	1.421 (3)
C4—H4A	0.9700	C17—C18	1.338 (3)
C4—H4B	0.9700	C17—H17	0.9300
C5—C6	1.586 (3)	C18—H18	0.9300
C5—H5A	0.9700	C19—H19A	0.9600
C5—H5B	0.9700	C19—H19B	0.9600
C6—C13	1.493 (3)	C19—H19C	0.9600
C6—C7	1.666 (3)		
			
C10—O1—H1	109.5	C12—C7—C6	128.71 (15)
C16—O2—H2	109.5	C7—C8—C9	119.95 (19)
C19—O3—H3	109.5	C7—C8—H8	120.0
C6—C1—C2	107.79 (16)	C9—C8—H8	120.0
C6—C1—H1A	110.1	C10—C9—C8	129.39 (16)
C2—C1—H1A	110.1	C10—C9—H9	115.3
C6—C1—H1B	110.1	C8—C9—H9	115.3
C2—C1—H1B	110.1	C11—C10—C9	112.22 (19)
H1A—C1—H1B	108.5	C11—C10—O1	117.78 (18)
C3—C2—C1	120.64 (15)	C9—C10—O1	129.99 (16)
C3—C2—H2A	107.2	C10—C11—C12	117.69 (19)
C1—C2—H2A	107.2	C10—C11—H11	121.2
C3—C2—H2B	107.2	C12—C11—H11	121.2
C1—C2—H2B	107.2	C7—C12—C11	131.45 (16)
H2A—C2—H2B	106.8	C7—C12—H12	114.3
C4—C3—C2	111.17 (16)	C11—C12—H12	114.3
C4—C3—H3A	109.4	C18—C13—C14	116.89 (18)
C2—C3—H3A	109.4	C18—C13—C6	117.07 (18)
C4—C3—H3B	109.4	C14—C13—C6	125.86 (15)
C2—C3—H3B	109.4	C15—C14—C13	127.54 (17)
H3A—C3—H3B	108.0	C15—C14—H14	116.2
C3—C4—C5	104.04 (16)	C13—C14—H14	116.2
C3—C4—H4A	110.9	C16—C15—C14	114.0 (2)
C5—C4—H4A	110.9	C16—C15—H15	123.0
C3—C4—H4B	110.9	C14—C15—H15	123.0
C5—C4—H4B	110.9	C15—C16—O2	114.96 (19)
H4A—C4—H4B	109.0	C15—C16—C17	119.83 (18)
C6—C5—C4	122.27 (14)	O2—C16—C17	125.21 (16)
C6—C5—H5A	106.8	C18—C17—C16	125.45 (17)
C4—C5—H5A	106.8	C18—C17—H17	117.3
C6—C5—H5B	106.8	C16—C17—H17	117.3
C4—C5—H5B	106.8	C13—C18—C17	116.28 (19)
H5A—C5—H5B	106.6	C13—C18—H18	121.9
C13—C6—C1	118.62 (15)	C17—C18—H18	121.9
C13—C6—C5	100.64 (16)	O3—C19—H19A	109.5
C1—C6—C5	106.23 (15)	O3—C19—H19B	109.5
C13—C6—C7	108.39 (14)	H19A—C19—H19B	109.5
C1—C6—C7	102.45 (15)	O3—C19—H19C	109.5
C5—C6—C7	121.61 (14)	H19A—C19—H19C	109.5
C8—C7—C12	109.31 (19)	H19B—C19—H19C	109.5
C8—C7—C6	121.98 (18)		
			
C6—C1—C2—C3	−53.3 (2)	C9—C10—C11—C12	0.5 (3)
C1—C2—C3—C4	54.9 (2)	O1—C10—C11—C12	179.62 (15)
C2—C3—C4—C5	−48.21 (19)	C8—C7—C12—C11	0.3 (3)
C3—C4—C5—C6	57.8 (2)	C6—C7—C12—C11	179.37 (17)
C2—C1—C6—C13	−64.5 (2)	C10—C11—C12—C7	−0.7 (3)
C2—C1—C6—C5	47.66 (18)	C1—C6—C13—C18	−17.5 (3)
C2—C1—C6—C7	176.22 (12)	C5—C6—C13—C18	−132.79 (19)
C4—C5—C6—C13	65.4 (2)	C7—C6—C13—C18	98.6 (2)
C4—C5—C6—C1	−58.8 (2)	C1—C6—C13—C14	167.53 (18)
C4—C5—C6—C7	−175.10 (15)	C5—C6—C13—C14	52.3 (2)
C13—C6—C7—C8	114.6 (2)	C7—C6—C13—C14	−76.4 (2)
C1—C6—C7—C8	−119.2 (2)	C18—C13—C14—C15	1.6 (3)
C5—C6—C7—C8	−1.0 (3)	C6—C13—C14—C15	176.5 (2)
C13—C6—C7—C12	−64.4 (2)	C13—C14—C15—C16	−0.5 (3)
C1—C6—C7—C12	61.8 (2)	C14—C15—C16—O2	179.16 (18)
C5—C6—C7—C12	179.96 (18)	C14—C15—C16—C17	−0.8 (3)
C12—C7—C8—C9	0.2 (3)	C15—C16—C17—C18	1.3 (3)
C6—C7—C8—C9	−179.03 (15)	O2—C16—C17—C18	−178.71 (19)
C7—C8—C9—C10	−0.2 (3)	C14—C13—C18—C17	−1.1 (3)
C8—C9—C10—C11	−0.2 (3)	C6—C13—C18—C17	−176.46 (17)
C8—C9—C10—O1	−179.14 (17)	C16—C17—C18—C13	−0.2 (3)

### Hydrogen-bond geometry (Å, °)

**Table:**

*D*—H···*A*	*D*—H	H···*A*	*D*···*A*	*D*—H···*A*
O1—H1···O2^i^	0.82	1.60	2.415 (2)	173
O2—H2···O3^ii^	0.82	2.08	2.860 (3)	159
O3—H3···O1^iii^	0.82	1.96	2.767 (2)	166

Symmetry codes: (i) *x*, *y*−1, *z*; (ii) *x*, *y*+1, *z*; (iii) *x*−1, *y*, *z*.
